# PrEP stigma among current and non-current PrEP users in Thailand: A comparison between hospital and key population-led health service settings

**DOI:** 10.3389/fpubh.2022.1019553

**Published:** 2022-12-02

**Authors:** Sineenart Chautrakarn, Ajaree Rayanakorn, Kannikar Intawong, Chonlisa Chariyalertsak, Porntip Khemngern, Scott Stonington, Suwat Chariyalertsak

**Affiliations:** ^1^Faculty of Public Health, Chiang Mai University, Chiang Mai, Thailand; ^2^Division of AIDS and STIs, Department of Disease Control, Ministry of Public Health, Nonthaburi, Thailand; ^3^Departments of Internal Medicine and Anthropology, University of Michigan, Ann Arbor, MI, United States

**Keywords:** PrEP stigma, HIV prevention, high-risk populations, ending AIDS, universal health coverage

## Abstract

**Background:**

Pre-exposure prophylaxis (PrEP) has demonstrated effectiveness in high-risk populations. PrEP service in Thailand became free of charge under the Universal Health Coverage (UHC) in 2021. The National Health Security Office launched a pilot project in 2020 to ensure sustainable service delivery, and the national monitoring and evaluation (M&E) framework was adopted to evaluate early phase implementation. We carried out a cross-sectional survey as part of the M&E process to investigate PrEP stigma among current and non-current PrEP users from both hospital and Key Population Led Health Services (KPLHS) settings in Thailand.

**Methods:**

Between August and October 2020, an online cross-sectional survey was conducted. A link for a self-administered questionnaire was distributed to all active PrEP centers and PrEP clients were then recruited by PrEP providers. Descriptive and univariate analysis using Chi-square were applied in the analyses. Attitudes toward PrEP were ranked from the most negative to the most positive. The negative attitude can be interpreted as PrEP stigma.

**Results:**

This study included 513 PrEP clients (355 from hospitals and 158 from KPLHS). In both settings, respondents' attitudes toward PrEP were generally positive, but some potential stigma was observed. 31.8% of hospital PrEP clients and 9.5% of KPLHS clients agreed that PrEP users should keep their pills hidden from others. Almost half (44.5%) of hospital clients and 18.4% of KPLHS clients agreed that PrEP users are often viewed negatively by society. More than 20% of hospital clients and 12% of KPLHS agreed that PrEP users frequently experience difficulties when their partner/lover/family find out that he or she is on PrEP. Respondents from the hospitals had slightly higher PrEP stigma than those from KPLHS.

**Conclusions:**

According to our findings, at the policy level, the campaign to provide PrEP education to all groups of people should be continued in order to promote a positive view of PrEP and reduce PrEP-related stigma among the general population, which is critical for successful PrEP implementation.

## Introduction

Pre-Exposure Prophylaxis (PrEP) is a proven effective strategy that involves daily oral administration of TRUVADA^®^ (emtricitabine/tenofovir disoproxil fumarate) to prevent HIV acquisition in high-risk populations (1–5). The World Health Organization (7) approved PrEP as an additional biomedical prevention strategy and issued PrEP use guidelines for specific groups of people, such as sero-discordant couples and men who have sex with men (MSM) (6). Recently, the WHO expanded the recommendation to include all groups at high risk of HIV infection (7). In addition, the CDC published clinical guidelines for administering PrEP to at-risk individuals in 2018 (8).

In Thailand, PrEP has been used as an HIV prevention strategy among high-risk populations since 2014. PrEP services have been run as a pilot program funded by both the public and private sectors, including the Joint United Nations Program on HIV/AIDS, the Thai Red Cross Princess Soamsawali HIV Prevention Program (known as “Princess PrEP Program”) and the Global Fund to Fight AIDS, Tuberculosis and Malaria (9). PrEP services in Thailand are available through hospitals by healthcare practitioners, and a network of community-based organizations, with PrEP clients able to visit key population-led health services (KPLHS) for medications, follow-up, and PrEP-related services. The KPLHS model was established in the country in 2015 in response to the needs of key populations at risk for HIV (10, 11). KPLHS are a defined set of HIV-related health services that focus on specific key populations and are delivered by community-based organizations (CBOs) run by those same key populations in collaboration with other health sector entities. In this context, community leadership means that the services necessary for addressing the HIV epidemic and related health issues are identified by the community itself and are, therefore, needs-based, demand-driven, and client-centered (12). These are designed and co-delivered by the KP community, in close collaboration with the public health sector, to ensure services are free from disrespectful care, verbal and physical abuse, and outright denial of care due to stigma and discrimination which often characterize conventional health care settings (10, 13, 14). This model has proven to be feasible and accessible in reaching high-risk individuals such as men who have sex with men (MSM), transgender women (TGW), sex workers (SWs), and people who inject drugs (PWID), who account for two-thirds of new HIV cases in Thailand (15).

PrEP service in Thailand became free of charge under the Universal Health Coverage (UHC) in 2021 and will be expanded the following years. To ensure sustainable service delivery, the National Health Security Office (NHSO) launched a pilot project in 2020 to provide oral TDF-based PrEP to 2,000 new clients (dubbed PrEP2000) at 49 PrEP service centers across the country in 21 provinces (46 hospitals and 3 KPLHS). In this regard, Thailand's National PrEP Program's monitoring and process evaluation (M&E) framework has been adopted to evaluate early phase implementation, which included success and barriers for the services.

PrEP-related stigma and shaming are potential barriers to PrEP implementation and maintenance (16–18). Stigma is defined as any attribute that marks a person as socially devalued (19). PrEP is potentially socially stigmatized because it is known to be the same medication taken by HIV-positive people, so it is stigmatized by association (20) with the assumption that HIV-positive people contracted the virus as a result of socially unacceptable behavior, such as promiscuity or injection drug use (20, 21). PrEP stigma can have significant negative consequences for PrEP adopters, such as suboptimal adherence, PrEP discontinuation, and a failure to disclose PrEP use to peers or a failure to disseminate PrEP information to other potential PrEP users (14, 16–18, 22). PrEP stigma can also have social and personal consequences for an individual's reputation and interpersonal relationships with friends, family, sexual partners, and healthcare providers (16, 23).

This cross-sectional survey was conducted as part of the M&E process to investigate PrEP stigma among current and non-current PrEP users from both hospital and KPLHS settings, as well as to compare PrEP stigma between the two settings. The findings will be useful in planning the national roll-out of the PrEP program under the country's UHC in order to maximize uptake and retention, with the WHO's and the country's ultimate goal of ending AIDS by 2030.

## Methods

### Participants

We performed an online based cross-sectional study between August and October 2020. The participants of this study were either current and non-current PrEP users from all high-risk populations, including heterosexuals, bisexuals, MSM, transgender people, pansexual lesbians, and others from active PrEP service centers. There was no sample size calculation. We used non-probability convenience sampling to recruit as large a sample of the key populations as possible.

### Data collection

The anonymous self-administered online survey was stored using a questionnaire QR code and link and distributed to all 49 active PrEP centers (46 hospitals and 3 KPLHS) across the country in 21 provinces. Clients who came to the clinic for PrEP counseling or who were already on PrEP and willing to participate in the study would then be recruited by the PrEP providers to complete a self-administered questionnaire.

Study subjects could access the study information sheet and informed consent form online. To participate in the study, the study participants were asked to provide informed consent by clicking on the screen at the first page of the online questionnaire, after which the questionnaire appeared.

The questionnaire was developed by the M&E research team with inputs from the national PrEP working committee under the Department of Disease Control, Ministry of Public Health.

This study was approved by the Research Ethics Committee, Faculty of Public Health, Chiang Mai University, Thailand (Document No. ET017/2020).

### Measurement and interpretation

PrEP stigma was assessed using eight items that included both negative and positive statements. The negative statement contained four items: “I am embarrassed to take PrEP in public.”, “PrEP users should keep their pills hidden from others.”, “People who take PrEP are often viewed negatively by society.”, and “People who use PrEP frequently experience difficulties when their partner/lover/family find out that he or she is on PrEP.” There were four positive statements: “I am proud to be able to take PrEP every day.”, “My friend/lover will encourage me to take PrEP.”, “My family will encourage me to take PrEP.”, and “People who use PrEP should be admired for taking responsibility for themselves.”

All respondents' attitudes were ranked from the most negative to the most positive. For negative statements, the responses were divided into three categories: negative attitude (agree and strongly agree), undecided, and positive attitude (strongly disagree and disagree). For positive statements, responses were divided into three categories: negative attitude (strongly disagree and disagree), undecided, and positive attitude (agree and strongly agree). The negative attitude can be interpreted as PrEP stigma.

### Statistical analysis

STATA version 16.1 (College Station, Texas, USA) was used for statistical analysis. Descriptive analysis was applied for demographic data of respondents overall and by type of service delivery model (Hospitals and KPLHS). Univariable analysis using Chi-square was employed to determine the difference of the proportion of self-rated PrEP stigma between hospital and KPLHS settings. The level of significance was set at *p* < 0.05.

## Results

### Respondent characteristics

This study included 513 PrEP clients from 24 hospitals and 3 KPLHS. There were 355 PrEP clients from the hospital and 158 from KPLHS. The mean age of respondents from hospitals was 30.81, while the mean age of respondents from KPLHS was 28.39. The majority of respondents' sex at birth from hospitals were male (*n* = 310, 87.3%) and from KPLHS were also male (*n* = 151, 95.6%). The majority of PrEP clients from both hospitals and KPLHS were MSM (60.8 and 79.1%, respectively). When the sociodemographic characteristics of the respondents from hospitals and KPLHS were compared, statistically significant differences in age, sex at birth, gender orientation, marital status, level of graduation, occupation and monthly income were found, as shown in Table 1.

**Table 1:** Respondent demographics (*n* = 513).

	**Overall (*n* = 513)**	**Hospital (*n* = 355)**	**KPLHS (*n* = 158)**	***p*-value**
**Age (year)**				0.012^*^
< 26	141 (27.5%)	89 (25.0%)	52 (32.9%)	
26–35	267 (52.0%)	180 (50.7%)	87 (55.1%)	
36–45	82 (16.0%)	68 (19.2%)	14 (8.9%)	
46 up	23 (4.5%)	18 (5.1%)	5 (3.1%)	
Mean (SD)	30.07 (7.629) Min = 14, Max = 71	30.81 (7.766) Min = 16, Max = 71	28.39 (7.048) Min = 14, Max = 62	0.001^**^
**Sex at birth**				0.004^*^
Male	461 (89.9%)	310 (87.3%)	151 (95.6%)	
Female	52 (10.1%)	45 (12.7%)	7 (4.4%)	
**Current gender/gender orientation**				< 0.001^*^
Heterosexual	94 (18.3%)	82 (23.1%)	12 (7.6%)	
Bisexual	46 (9.0%)	31 (8.7%)	15 (9.5%)	
MSM	341 (66.5%)	216 (60.8%)	125 (79.1%)	
Transgender people	26 (5.1%)	22 (6.2%)	4 (2.5%)	
Others (lesbian, pansexual etc.)	6 (1.2%)	4 (1.2%)	2 (1.3%)	
**Marital status**				0.006^*^
Single	315 (61.4%)	201 (56.6%)	114 (72.2%)	
Couple	189 (36.8%)	147 (41.4%)	42 (26.6%)	
Divorced/Widowed	9 (1.8%)	7 (2.0%)	2 (1.2%)	
**Level of graduation**				0.011^*^
Less than bachelor degree	226 (44.1%)	172 (48.5%)	54 (34.2%)	
Bachelor	242 (47.2%)	154 (43.4%)	88 (55.7%)	
Master up	45 (8.8%)	29 (8.1%)	16 (10.1%)	
**Occupation**				0.049^*^
Student	75 (14.6%)	46 (13.0%)	29 (18.4%)	
Government official /Government employee	75 (14.6%)	60 (16.9%)	15 (9.5%)	
Self-employed	90 (17.5%)	55 (15.5%)	35 (22.2%)	
Office workers	244 (47.6%)	173 (48.7%)	71 (44.9%)	
Unemployed	29 (5.7%)	21 (5.9%)	8 (5.1%)	
**Monthly income (THB)**				0.010^*^
< 5,000	53 (10.3%)	40 (11.3%)	13 (8.2%)	
5,000–9,999	88 (17.2%)	71 (20.0%)	17 (10.8%)	
10,000–14,999	96 (18.7%)	68 (19.2%)	28 (17.7%)	
15,000–19,999	99 (19.3%)	71 (20.0%)	28 (17.7%)	
20,000–29,999	89 (17.3%)	52 (14.6%)	37 (23.4%)	
30,000 up	88 (17.2%)	53 (14.9%)	35 (22.2%)	

^*^Significant level using Chi-Square test.

^**^Significant level using Independent *T*-test.

In terms of PrEP client category, the majority of hospital respondents were clients who continued taking PrEP (63.7%) and followed by newly offered and agreed to initiate PrEP (20.3%), whereas the majority of KPLHS respondents were people who continued using PrEP (57.0%) and followed by newly offered but refused to start (19.0%) and PrEP discontinuous due to row risk behaviors or do not want to continue taking PrEP for any reason (17.7%). There were statistically significant differences in client categories between hospital and KPLHS settings (Table 2).

**Table 2:** PrEP client category (*n* = 513).

**PrEP client category**	**Overall (*n* = 513)**	**Hospital (*n* = 355)**	**KPLHS (*n* = 158)**	***p*-value**
Newly offered and initiated	82 (16.0%)	72 (20.3%)	10 (6.3%)	< 0.001^*^
Newly offered but refused to start	60 (11.7%)	30 (8.4%)	30 (19.0%)	
PrEP continuing	316 (61.6%)	226 (63.7%)	90 (57.0%)	
PrEP discontinuous (both at the clinic and by themselves)	55 (10.7%)	27 (7.6%)	28 (17.7%)	

^*^Significant level using Chi-Square test.

### PrEP stigma

The findings revealed that overall attitudes toward PrEP were quite positive in both settings. More than 70% of respondents expressed a positive attitude toward the following statements: “I am proud to be able to take PrEP every day.”, “My friend/lover will encourage me to take PrEP.”, and “People who use PrEP should be admired for taking responsibility for themselves.” However, we discovered that only 60% of hospital respondents and 52.5% of KPLHS respondents responded positively to the statement “My family will encourage me to take PrEP.” This meant that nearly half of respondents disagreed or were unsure whether their family encouraged them to use PrEP.

**Figure 1:**
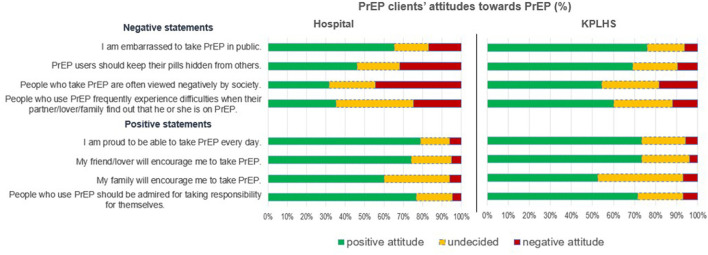
PrEP clients' attitudes toward PrEP, PrEP stigma among current and non-current PrEP users in Thailand: A comparison between hospital and key population-led health service settings, Thailand 2022.

In terms of negative statements, despite the fact that a minority of respondents reported negative attitudes toward PrEP, which can be interpreted as PrEP stigma, some potential stigma was observed in these findings. One-third of respondents from hospital agreed with statement “PrEP users should keep their pills hidden from others.”, and nearly half of them agreed that “People who take PrEP are often viewed negatively by society.” When compared to KPLHS, these differences were statistically significant (*p*-value 0.001) (9.5 and 18.4%, respectively). More than 20% of hospital respondents and 12% of KPLHS agreed with the statement “People who use PrEP frequently experience difficulties when their partner/lover/family find out that he or she is on PrEP.” This difference was statistically significant (*p*-value < 0.001). For the statement “I am embarrassed to take PrEP in public.”, 16.9% of hospital respondents agreed with it whereas only 6.3% of KPLHS respondents agreed, and this difference was statistically significant (*p*-value 0.005). Respondents from the hospitals had slightly higher PrEP stigma than those from KPLHS (Table 3 and Figure 1).

**Table 3:** Respondents' attitudes toward PrEP (*n* = 513).

	**Hospital (*****n*** = **355)**			**KPLHS (*****n*** = **158)**			
**Negative statements**	**Disagree (positive attitude)**	**Undecided**	**Agree (negative attitude)**	**Disagree (positive attitude)**	**Undecided**	**Agree (negative attitude)**	***p*** **-value**
I am embarrassed to take PrEP in public.	232 (65.4%)	63 (17.7%)	60 (16.9%)	120 (76.0%)	28 (17.7%)	10 (6.3%)	0.005^*^
PrEP users should keep their pills hidden from others.	164 (46.2%)	78 (22.0%)	113 (31.8%)	109 (69.0%)	34 (21.5%)	15 (9.5%)	< 0.001^*^
People who take PrEP are often viewed negatively by society.	112 (31.6%)	85 (23.9%)	158 (44.5%)	86 (54.4%)	43 (27.2%)	29 (18.4%)	< 0.001^*^
People who use PrEP frequently experience difficulties when their partner/lover/family find out that he or she is on PrEP.	125 (35.2%)	142 (40.0%)	88 (24.8%)	95 (60.1%)	44 (27.9%)	19 (12.0%)	< 0.001^*^
**Positive statements**	**Agree (positive attitude)**	**Undecided**	**Disagree (negative attitude)**	**Agree (positive attitude)**	**Undecided**	**Disagree (negative attitude)**	***p*** **-value**
I am proud to be able to take PrEP every day.	280 (78.9%)	54 (15.2%)	21 (5.9%)	116 (73.4%)	33 (20.9%)	9 (5.7%)	0.286
My friend/lover will encourage me to take PrEP.	263 (74.1%)	74 (20.8%)	18 (5.1%)	116 (73.4%)	36 (22.8%)	6 (3.8%)	0.749
My family will encourage me to take PrEP.	213 (60.0%)	121 (34.1%)	21 (5.9%)	83 (52.5%)	64 (40.5%)	11 (7.0%)	0.287
People who use PrEP should be admired for taking responsibility for themselves.	272 (76.6%)	67 (18.9%)	16 (4.5%)	113 (71.5%)	34 (21.5%)	11 (7.0%)	0.364

^*^Significant level using Chi-Square test.

## Discussion

To the best of our knowledge, this is preliminary data to provide attitudes toward PrEP and PrEP stigma among hospital and KPLHS PrEP clients from PrEP service centers across Thailand. These findings have significant implications for the design and implementation of PrEP services under Universal Health Coverage. PrEP clients from both service models have distinct characteristics. The majority of KPLHS clients were MSM, who may be comfortable or familiar with services that respond to the needs of key populations at risk for HIV and are run by those same key populations, such as MSM or TGW (10–12). Although the proportion of PrEP newly offered for both models of services was quite similar (28.8% from hospitals and 25.3% from KPLHS), those from hospitals agreed to initiate a much higher proportion than those from KPLHS (20.3 and 6.3%). A possible cause of this observation is that the majority of providers at the hospital are health care professionals (9), and by nature, patients or clients may respect and follow the advice of health care professionals (24).

In the current study, the majority of PrEP clients from both service models had positive attitudes toward PrEP in terms of being proud to take PrEP for self-protection and receiving support from family and friends for taking PrEP. These mean that PrEP is now widely known and recognized for its benefits in HIV self-protection. However, some potential PrEP stigma attitudes were identified in this study. Nearly half of hospital PrEP clients and nearly 20% of KPLHS PrEP clients agreed that PrEP users are frequently viewed negatively by society. This might be linked to HIV stigma because PrEP is HIV medication, and it is known to be the same medication taken by HIV-positive people, so it is stigmatized by association (20, 21). People's perceptions or misconceptions lead them to believe PrEP and ART are the same, especially if they do not recognize the brand or generic names and only see similar ingredient lists, so PrEP medication may cause HIV-related stigma and/or fear in its users. Recent studies have revealed the same finding, that PrEP stigma is linked to HIV stigma (16, 22, 25–30). In addition, this study found one-third of hospital PrEP clients agreed that PrEP users should keep their pills hidden from others and nearly 20% of them also agreed that they are embarrassed to take PrEP in public. This may mean that PrEP users were ashamed of their PrEP use; this stigma may also be linked to HIV stigma.

In addition, about 25% of PrEP clients from hospital and 12% from KPLHS agreed that people who use PrEP frequently experience difficulties when their partner/lover/family find out that he or she is on PrEP. These findings were similar to those found in studies of transgender women in the United States (22), MSM in the United States (16, 26, 31), women in the United States (29, 32), and young people in Uganda, Zimbabwe, and South Africa (25). This demonstrates that some members of society have negative attitudes toward PrEP and may object if close relatives use it.

Furthermore, it should be noted that in the current study, some PrEP clients did not decide whether they agreed or disagreed with all of the PrEP stigma questions. This could be because they did not have specific ideas about PrEP at the time. In this case, these people may or may not face PrEP stigma in the future. As a result, the campaign to educate people about PrEP should be continued in order to promote a positive view of PrEP among key populations and the general population, as well as to provide training support for HIV-related health workers and lay health workers, because training has been shown to be one of the most effective intervention strategies for supporting HIV pre-exposure prophylaxis (PrEP) implementation (33).

When comparing PrEP stigma across settings, it appeared that people who received PrEP service at a hospital had a higher stigma than those who received service at KPLHS. This could be because at the hospital, PrEP services are typically delivered by healthcare professionals and integrated with ARV, STI clinics, and VCT, active client recruitment, and mobile VCT, all of which have such a high workload that they may be unable to provide adequate time for counseling for every patient. This is the distinction between the two service delivery models, as evidenced by client negative attitudes toward PrEP, which appear to be low among KPLHS clients. as the context of the KPLHS, which was established in response to the needs of key populations at risk of HIV and is run by those same key populations. This model demonstrates that it is feasible, acceptable, and affordable, while also expanding service delivery options for those in need (10). However, the country currently has a small number of KPLHS located in major cities such as Bangkok, Chonburi, Chiang Mai, and Songkhla (12). According to the evidence from the current study, the country should increase the number of KPLHS across the country, as well as their capacity, in order to provide an alternative for clients and alleviate the burden of this work in the hospitals.

Over the last decade, Thailand has successfully reduced its HIV epidemic, with the total number of annual new infections trending downward (34–36). In addition, Thailand became the first Asian country to meet the World Health Organization's (WHO) targets for eliminating mother-to-child transmission (MTCT) in 2015 (37–39). Although, Thailand's overall progress, not all key populations such as MSM and TGW reflect the same improvement. From 2014 to 2018, MSM, TGW, IDU, and sex workers were responsible for 46.2% of new infections in Thailand (36). To achieve the country's ultimate goal of eradicating AIDS by 2030, a variety of strategies, including increased screening for early treatment and prevention, are required to pave the way. One of these strategies is PrEP service, which will be provided free of charge under the country's Universal Health Coverage (UHC) in 2021. The challenges in providing PrEP services include a lack of awareness about PrEP among both HCPs and potential PrEP clients, a high workload, limited manpower, counseling space, and coverage of the benefit package under UHC (9). It should be noted that HCPs' attitudes toward PrEP play an important role in PrEP service delivery. Negative attitudes toward PrEP are a major impediment to delivering PrEP to clients (40–43). Furthermore, the stigma and shaming associated with PrEP are significant barriers to PrEP initiation and maintenance. As a result, interventions aimed at reducing PrEP-related stigma and promoting a positive view of PrEP among key populations and the general population, as well as in the health care providers should be implemented.

## Limitations

The current study was part of the national monitoring and evaluation (M&E) planned for evaluating the early phase of PrEP service implementation in the country, and it is the first national survey involving PrEP clients from PrEP centers in both hospital and KPLHS settings. As a result, the findings of this study seem to provide a good representation of PrEP clients in Thailand. However, there are some limitations to our study. First, we were unable to calculate the response rate because the online survey was distributed to each PrEP center *via* QR code and link, and PrEP providers recruited their clients to participate in the study, and there may have been selection bias during recruitment. Second, there is limited comparability with other surveys because the questionnaire was developed by the M&E research team with input from the national PrEP working committee of the Ministry of Public Health's Department of Disease Control, as well as limited comparability with other surveys using a variety of questions and methodologies. Third, because the purpose of this study was to investigate and compare PrEP stigma among current and non-current PrEP users from both hospital and KPLHS settings. We could not rule out the possibility that differences in demographics or PrEP client categories played a role in stigma. Fourth, because these analyses were cross-sectional in nature, we cannot draw any conclusions about the causes of the observed differences in PrEP attitudes. Finally, because this was a questionnaire-based study, respondents were unable to express any other opinions or attitudes toward PrEP. As a result, the findings may not accurately reflect other PrEP stigma attitudes that this study did not capture.

## Conclusion

Despite the fact that the majority of PrEP clients had positive attitudes toward PrEP, this study identified some potential PrEP stigma. According to these findings, at the policy level, the campaign to provide PrEP education to all groups of people should be continued in order to promote a positive view of PrEP and reduce PrEP-related stigma among the general population, which is critical for successful PrEP implementation.

## Data availability statement

The raw data supporting the conclusions of this article will be made available by the authors, without undue reservation.

## Ethics statement

The studies involving human participants were reviewed and approved by the Research Ethics Committee, Faculty of Public Health, Chiang Mai University. Written informed consent for participation was not required for this study in accordance with the national legislation and the institutional requirements.

## Author contributions

SuC conceptualized, designed, and supervised the study. SiC, KI, and AR contributed to the design of the study and questionnaire. SiC, KI, CC, and PK organized the data collection. SiC carried out the analysis, wrote up the results, drafted, wrote the article, revised, and finalized the article. SuC and SS reviewed and commented on the draft of article. All authors contributed to the article and approved the submitted version.

## Funding

This work was supported by Global Fund to Fight AIDS, Tuberculosis, and Malaria, the Department of Disease Control, the Ministry of Public Health, and the Joint United Nations Programme on HIV/AIDS; UNAIDS.

## Conflict of interest

The authors declare that the research was conducted in the absence of any commercial or financial relationships that could be construed as a potential conflict of interest.

## Publisher's note

All claims expressed in this article are solely those of the authors and do not necessarily represent those of their affiliated organizations, or those of the publisher, the editors and the reviewers. Any product that may be evaluated in this article, or claim that may be made by its manufacturer, is not guaranteed or endorsed by the publisher.
